# Whole-brain responses to visual and auditory stimuli in anesthetized and minimally restrained awake mice using quiet zero echo time fMRI

**DOI:** 10.1162/imag_a_00384

**Published:** 2024-12-05

**Authors:** Lenka Dvořáková, Petteri Stenroos, Raimo A. Salo, Ekaterina Paasonen, Heikki Tanila, Shalom Michaeli, Silvia Mangia, Tamara Zehnder, Thomas Mueggler, Basil Künnecke, Jaakko Paasonen, Olli Gröhn

**Affiliations:** A.I. Virtanen Institute for Molecular Sciences, University of Eastern Finland, Kuopio, Finland; Neurocenter, Kuopio University Hospital, Kuopio, Finland; Center for Magnetic Resonance Research, University of Minnesota, Minneapolis, MN, United States; Neuroscience and Rare Diseases, Roche Pharma Research and Early Development, Roche Innovation Center Basel, Basel, Switzerland

**Keywords:** awake mouse fMRI, visual pathway, auditory pathway, zero echo time fMRI, sensory-evoked fMRI, ketamine–xylazine anesthesia

## Abstract

Functional MRI (fMRI) is a flexible tool for sensory perception studies in animal models. However, animal fMRI studies are generally performed under anesthesia. Unfortunately, anesthesia affects brain function and sensory processing, complicating the interpretation of the findings. Since there is a growing need for fMRI protocols applicable for awake animals, we optimized a zero echo time Multi-Band Sweep Imaging with a Fourier Transformation (MB-SWIFT) fMRI approach for imaging awake mice. We implemented a 14-day habituation protocol that resulted in merely moderate motion of the mice while being head-fixed with the animals’ body and limbs being free to move. The sensory responsiveness between different states of consciousness was compared by imaging mice with visual and auditory stimulation schemes in the awake state and under ketamine–xylazine anesthesia. In awake mice, we observed a robust whole-brain activation of the ascending auditory and visual pathways, as well as higher sensory processing areas. Under ketamine–xylazine anesthesia, auditory responses were suppressed, and the temporal shapes of fMRI responses were different from those obtained in awake mice. Our results suggest that the quiet and motion-tolerant zero echo time MB-SWIFT approach allows complex behavioral fMRI designs in the awake state that promise to improve our understanding of the underlying mechanisms of perception.

## 1 Introduction

Understanding sensory perception is a key objective in neuroscience, since it will contribute significantly to our comprehension of cognitive behavior (Schaffner et al., 2023). Mouse models have emerged as valuable tools for investigating visual and auditory sensory systems (Huberman & Niell, 2011;Keithley et al., 2004;Townsend et al., 2020). While the current standard methods, such as electrophysiological recordings (Postal et al., 2022), multiphoton microscopy (Klioutchnikov et al., 2022;Yildirim et al., 2019), and other optical techniques (White et al., 2011), have provided insights into the function of the sensory systems, they are limited to local signals and often require invasive procedures to gain access to the brain. Functional magnetic resonance imaging (fMRI) is a technique that can achieve noninvasive whole-brain mapping of the sensory pathways with its findings easily translatable to clinical research (Martin, 2014). Echo planar imaging (EPI) is commonly employed in fMRI as it can reach a high enough temporal resolution to detect the hemodynamic changes occurring near to the neuronal activity. However, the EPI technique suffers from several limitations; in particular, B_0_field inhomogeneities severely affect the image quality, while the rapidly switching gradients produce very loud acoustic noise. The sensitivity for B_0_distortions poses technical challenges particularly in mouse fMRI due to the small size of the animal’s brain. The images are, therefore, highly vulnerable to the image distortions created by the susceptibility gradients present at the tissue–bone interface, or around the air cavities of the ear canals. Consequently, these distortions can lead to significant signal losses in several important regions of interest. Furthermore, anesthesia has been routinely applied for fMRI measurements to alleviate stress and motion during data acquisition. Given that anesthetics affect hemodynamic responses (Schlegel et al., 2015), brain function (Paasonen et al., 2018), and sensory processing (Olcese et al., 2018), it is evident that the impact of anesthesia on evoked fMRI responses should be fully characterized and clarified.

Due to the aforementioned challenges, rather few investigators have exploited fMRI techniques to explore the murine visual system (Dinh et al., 2021;Hike et al., 2023;Niranjan et al., 2016;Zeng et al., 2022). Of these, onlyDinh et al. (2021)directly compared visual stimulation responses under anesthesia and wakefulness, observing differences in both regional response amplitudes and temporal characteristics between ketamine–xylazine-anesthetized and awake mice. Additionally, the majority of the previous studies had a rather limited coverage of the brain, primarily focusing on the posterior part of the brain, where the visual pathway areas are located. A recent study conducted byHike et al. (2023)included frontal areas in their field of view and, interestingly, observed an activation in the anterior cingulate area, emphasizing the need for full brain investigations for even simple sensory stimuli.

Auditory stimuli in fMRI present a far greater challenge due to the intense acoustic noise generated by the traditional EPI sequence. In an attempt to overcome this obstacle, a few research groups have delivered the auditory stimuli directly to the mouse’s ear canal (Blazquez Freches et al., 2018;Chen et al., 2020). These studies have yielded mixed results:Chen et al. (2020)reported strong cortical responses to the auditory stimuli in awake mice, whileBlazquez Freches et al. (2018)observed no cortical responses in anesthetized mice. These findings underscore the significance of clarifying the impact of anesthesia on auditory processing. Nonetheless it should be emphasized that investigating auditory responses is not straightforward with conventional loud fMRI techniques, highlighting the need for improved methodologies.

Recently, zero echo time fMRI has emerged as a promising alternative to the conventional EPI approach. We have previously shown that a zero echo time variant, Multi-Band Sweep Imaging with Fourier Transformation (MB-SWIFT) (Idiyatullin et al., 2015), is inherently motion tolerant, and the average sound pressure level is up to 20 dB lower than with the EPI (Paasonen et al., 2020). These features make MB-SWIFT ideal for behavioral rodent imaging, where body motion is expected or even desired as a presentation of behavior. While a head-fixed setup with minimal body restraint has been used before in rats with MB-SWIFT (Paasonen et al., 2022), so far it has not been implemented in mice. Hence, the aims of this study were twofold: first to demonstrate the feasibility of undertaking zero echo time fMRI in mice and second, to address the obstacles in the recent mouse sensory fMRI studies, where the brain coverage has often been limited, and the impact of anesthesia has been underexplored. Initially, we developed a novel fMRI approach suitable for use in minimally restrained awake mice, and then validated the performance of the method by studying fMRI responses to visual and auditory stimuli. Finally, the results from awake mice were compared with those obtained under ketamine–xylazine anesthesia, a technique commonly used in rodent experiments.

## 2 Methods

### 2.1 Animals and habituation

**Fig. 1.**
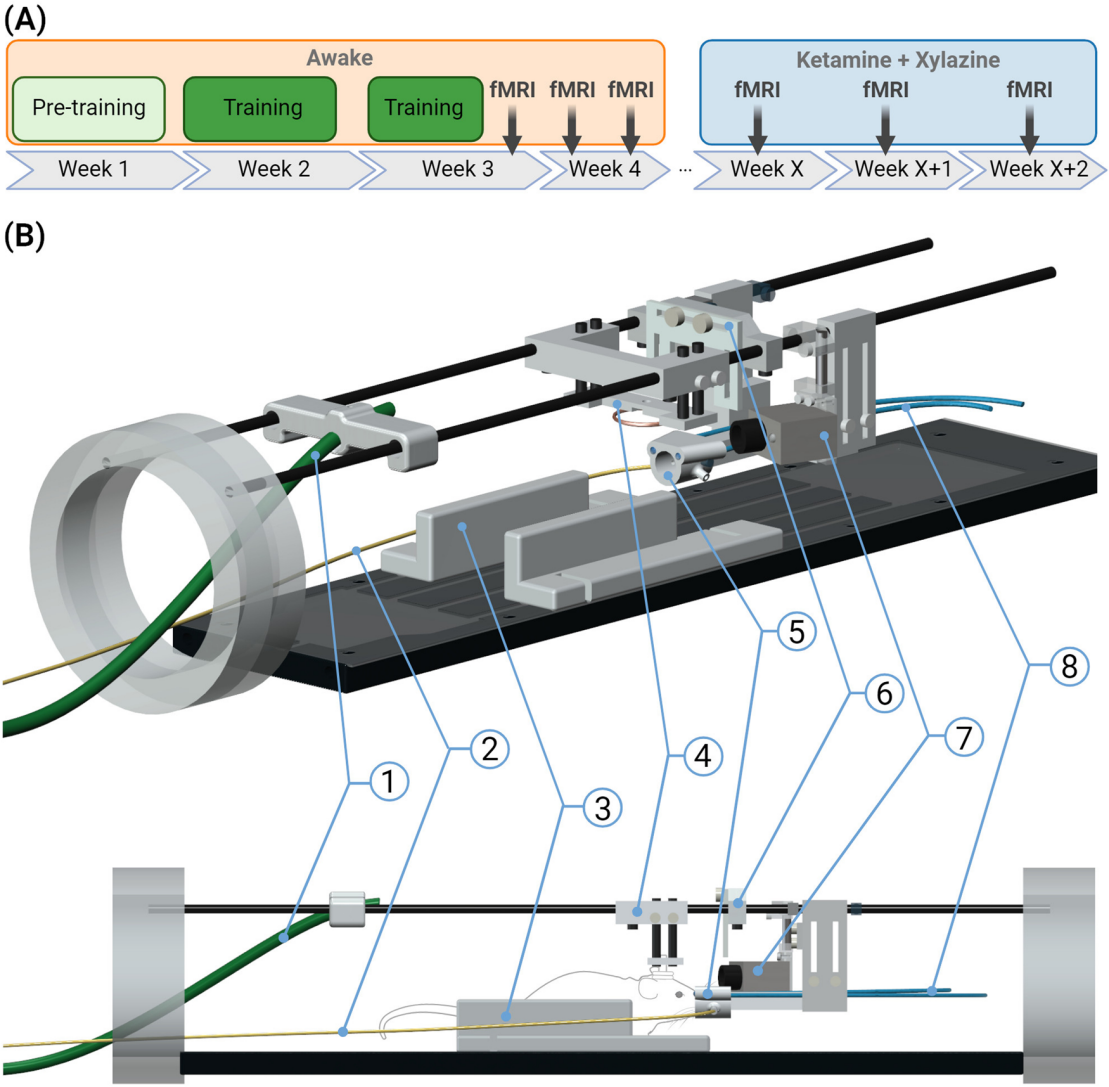
(A) Timeline of the training and the fMRI experiments, where the time between the awake and anesthetized experiments X = 3–9 weeks. (B) A visualization of the MRI holder with the delivery systems for auditory and visual stimuli. (1) Tube for the auditory stimuli; (2) anesthesia/gas tube; (3) 3D printed walls to limit sideways motion of the animal; (4) 3D printed head restraint holder that fits the headpost; (5) 3D printed nose cone for the delivery of anesthesia/gas with built-in holders for the optical fibers; (6) 3D printed holder for radiofrequency coil; (7) camera; (8) optical fibers for the visual stimulation. Partially created inhttps://www.BioRender.com.

Animal procedures were approved by the Finnish Animal Experiment Board (licence numbers: ESAVI-2023-19957 OH3 and ESAVI-2019-028408). In this study, C57BL/6 mice (4 males and 4 females; aged 8–12 months) were used. The animals were individually housed and maintained on a 12/12 h light–dark cycle. Food and water were available*ad libitum*. All mice underwent surgery, where a headpost designed to fit to the animal holder for awake fMRI (Fig. 1) was implanted. Briefly, the mouse was anesthetized with isoflurane (5% induction with 1.8–2.2% maintenance at the same N2/O2 70%/30% mixture) and then the skull was exposed. A headpost made from either polytetrafluoroethylene (PTFE) or polychlorotrifluoroethylene (PCTFE) was secured on the cleaned skull with a layer of dental cement. Carprofen (Rimadyl, Zoetis Finland Oy, 5 mg/kg s.c.) was given to relieve any postsurgical pain. After a minimum of 3 weeks of recovery, the animals underwent a habituation protocol (Supplementary Table 1) to mitigate stress and excessive motion in the subsequent awake fMRI procedures. We adapted a protocol previously described for rats (Paasonen et al., 2022). Here, the protocol consisted of 14 days of habituation over the course of 3 weeks, divided into two phases: pretraining and training. In the pretraining phase, animals were gradually acclimated to the handler, imaging holder, and the sound of the fMRI sequence. The animals were also trained to be restrained by hand holding of the headpost for short intervals of time. In the training phase, the animals were initially anesthetized with isoflurane and then head fixed in the holder while the rest of the body and legs were freely movable on top of a semislippery glass or acrylic glass surface. The training time and the fMRI sound level were gradually increased with earplugs being used to protect the hearing. Additionally, during the last 3 days of the habituation, visual and auditory stimuli were applied with a nearly similar stimulation design as later would be used in the imaging sessions. Mice were given a treat (either Nutella or 1% sucrose water, based on their preference) as a positive reinforcement before and after each training and measurement session.

### 2.2 Anesthesia protocol

In the fMRI experiments conducted under anesthesia, the animals were initially anesthetized with isoflurane (gradually increased to 3% in N2/O2 70%/30% mixture) and then injected with a single dose of ketamine–xylazine (ketamine 100 mg/kg, xylazine 10 mg/kg, i.p.). A low level of isoflurane (0.5%) was maintained while inserting the earplugs, positioning the animal in the holder, and adjusting the optical cables for visual stimulation. During the scanning, breathing was monitored with a pressure sensor placed under the animal. The average breathing rate in the animals was 214 ± 26 breaths per minute. The temperature was measured with a rectal probe and kept stable (~37°C) with a warm water circulation system. After the measurement, the animals were allowed to recover from the anesthesia in their cages, which were kept on a heating pad to ensure a normal core temperature.

In the awake measurements, the animals were initially anesthetized with isoflurane (5% in N2/O2 70%/30% mixture), and a moderate level of isoflurane (1.6–2.0%) was maintained while inserting the earplugs, positioning the animal in the holder, adjusting the optic cables for the visual stimulation, the preparation scans (scout images and manual shim), and anatomical scans. The animal’s breathing rate and behavior were monitored with an MRI-compatible video camera with infrared light (12 M-i, MRC Systems GmbH, Heidelberg, Germany). The breathing was estimated from 10-s motion-free periods and the average observed respiration rate was 113 ± 29 breaths per minute. After the anatomical scans, the level of isoflurane was lowered to 0%. The flow of the N2/O2 mixture into the nose cone was kept constant to prevent the influence of isoflurane potentially lingering in the magnet bore. The functional scans were started once the animals showed signs of being awake (whisker or limb movement, typically after 2–3 min). At the end of the measurement, the animals were gradually anesthetized with isoflurane and removed from the MRI holder.

### 2.3 Magnetic resonance imaging

The timeline of the habituation and imaging is shown inFigure 1. All imaging was performed with a 9.4T magnet (Agilent DirectDrive console, Palo Alto, CA, USA) with a custom-made surface loop coil (inner diameter 11 mm on the short axis, 13 mm on the long axis; Neos Biotec, Pamplona, Spain) for both signal excitation and reception. The coil was fitted around the headpost. The aim was to perform functional imaging in all animals with three repetitions in both the awake and ketamine–xylazine anesthesia conditions. However, 2 animals were found to have died in their cages between the measurements, resulting in 23 awake sessions and 20 anesthesia sessions.

For the functional imaging, we implemented a radial 3D MB-SWIFT sequence with the following parameters: 2,047 spokes per spiral, 2 RF pulses per spoke, repetition time of 0.82 ms for a spoke, acquisition time of 1.7 s per volume, flip angle of 3°, excitation/acquisition bandwidths of 125/500 kHz, matrix size of 64 × 64 × 64, with 24 × 24 × 24 mm³ field of view, and isotropic resolution of 375 μm. Altogether 710 volumes were acquired in each fMRI scan, leading to an acquisition time of 20 min.

**Fig. 2.**
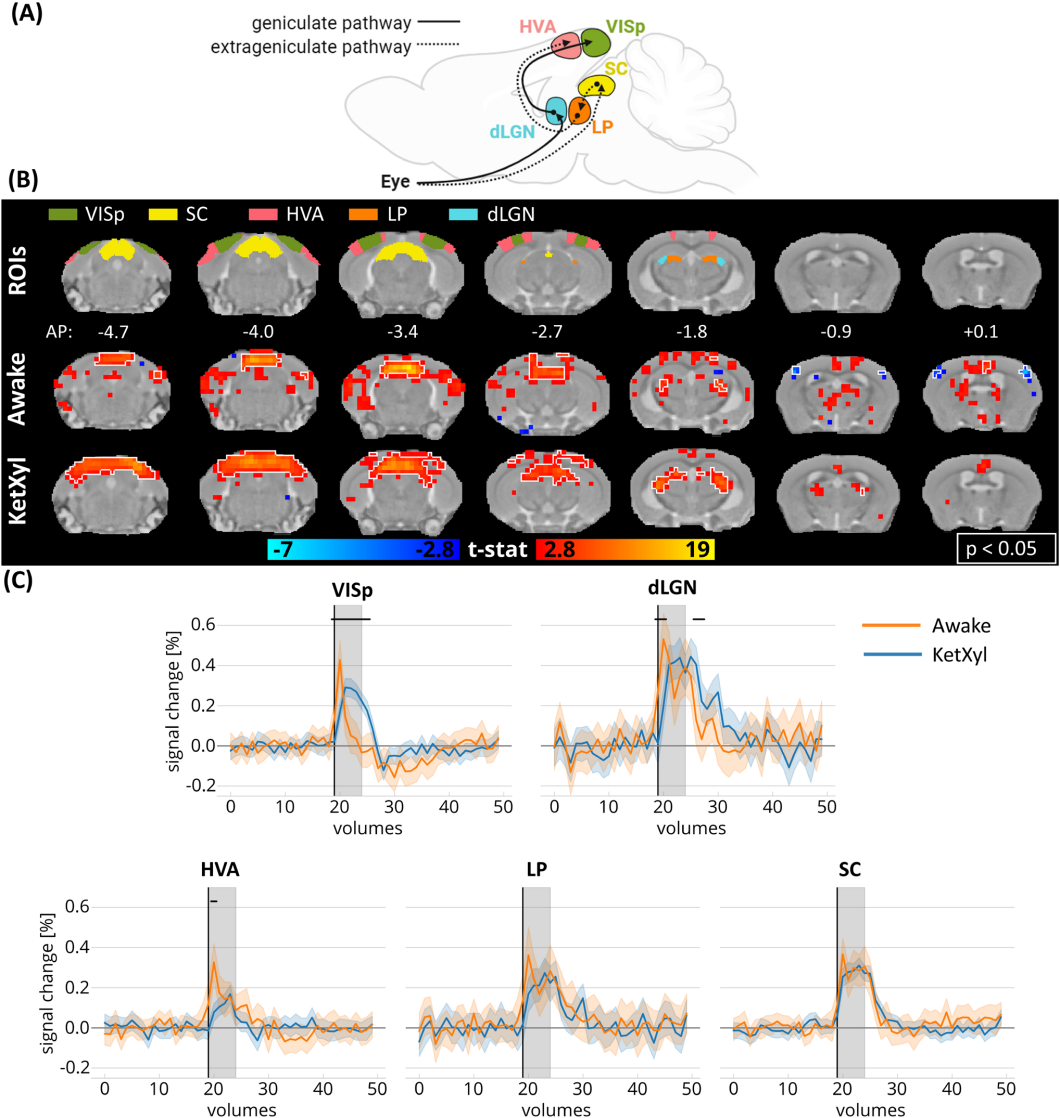
Activation maps and the time courses following visual stimulation in awake and anesthetized animals in visual pathway-relevant areas. (A) A simplified illustration of two visual pathways in mice. (B) Regions of interest (ROIs) in the areas of the visual pathways and the group activation maps in awake (n = 23 measurements) and anesthetized animals (n = 20 measurements) in selected slices related to the visual pathways (white outline indicates p < 0.05, family-wise error corrected). (C) Mean time courses in the VISp, dLGN, HVA, LP, and SC in awake and anesthetized animals. Horizontal black lines designate volumes where the two time series differed (independent volume-wise t-test, p < 0.05, FDR corrected, tested in volume numbers 19–30). Shaded plot: 95% confidence interval, the black vertical line marks the beginning of the stimulation and gray regions show the duration of the stimulus. dLGN, dorsal lateral geniculate nucleus; HVA, higher-order visual areas; LP, lateral posterior thalamic nucleus; SC, superior colliculus; VISp, primary visual cortical area. Partially created inhttps://www.BioRender.com.

Anatomical reference images were acquired with an MB-SWIFT sequence with the following parameters: 4,000 spokes per spiral, 16 stacks of spirals, repetition time of 3 ms for a spoke, flip angle of 4°, excitation/acquisition bandwidths of 192/384 kHz, matrix size of 256 x 256 x 256 voxels, 24 x 24 x 24 mm^3^field of view, and 93.75 µm isotropic resolution. To increase the anatomical contrast, a magnetization transfer pulse (sinc-shaped pulse, γB_1_125 Hz, offset 2,000 Hz, pulse duration 20 ms) was given every 32 spokes. The sequence had a total acquisition time of 4 min. The representative anatomical and fMRI images are shown inSupplementary Figures 1 and 2, respectively.

### 2.4 Visual and auditory stimulation experimental design

During imaging, light and/or sound stimulation were applied in a 10s-on-50s-off pattern. A stimulus generator (STG4008-16mA, Multi Channel Systems MCS GmbH, Reutlingen, Germany) with MC_Stimulus II software (version 3.5.11) was used to control stimulations with TTL pulses. For the light stimulation, a custom-built TTL-controlled Arduino-based stimulator was utilized, delivering 50 ms flashes of blue light (470 nm) at a frequency of 5 Hz during the fMRI. The light was guided forward via two optic cables to the top of a custom-built nose cone (Fig. 1). The nose cone was adjusted such that each eye was at the center of the illuminated area, ensuring a balanced light intensity for both eyes. For sound stimulation, we used pressurized air (2 bar) gated by a TTL-controlled solenoid valve (Neos Biotec, Pamplona, Spain). The sound stimulation consisted of a series of 5-ms air-pressure-induced sound pulses at a frequency of 13 Hz. Bench tests indicated that the spectrum of the air-puff-induced sound included a wide range of frequencies, resembling white noise. Sound pressure levels (SPL) inside the magnet bore were measured during auditory stimulation, and MB-SWIFT and EPI acquisition with an omnidirectional condenser microphone (MT830R, Audio-Technica Limited, Leeds, UK). The average SPL of the air puffs was 25 dB above the background noise, whereas the SPL of MB-SWIFT sequence averaged 13 dB above the background noise. For comparison, the noise of a standard EPI sequence was measured. The average SPL of the EPI noise was at least 43 dB above the background, as the peak SPL amplitudes of the sequence exceeded the maximum dynamic range of the microphone used (>46 dB above background noise). The pressurized air was delivered via a plastic tube, which was attached in the middle of the MRI holder to ensure equal stimulation of both ears. The ending of the tube was placed approximately 15 cm from the head of the animal to avoid stimulating its whiskers (Fig. 1). In order to eliminate lingering anesthesia effects in the awake measurements, the stimulation paradigm was initiated 5 min after the start of the fMRI sequence. A total of 15 stimuli were applied during the fMRI session: 5 visual, 5 auditory, and 5 simultaneous visual and auditory stimuli. The order of the stimuli was randomized for each measurement session to prevent adaptation effects from automatic sensory prediction arising from repeated exposure. Here we analyzed solely the single stimuli (i.e., either auditory or visual), resulting in five auditory and five visual stimuli per session.

### 2.5 fMRI data analysis

The MB-SWIFT MRI data were reconstructed using RF-pulse deconvolution, with a gridding and iterative FISTA algorithm (Beck & Teboulle, 2009) volume-by-volume with three iterations. All MRI data were processed and analyzed with in-house created scripts, Snakemake (https://snakemake.github.io/,Köster & Rahmann, 2012), Python (version 3.10,https://www.python.org/downloads/), advanced normalization tools (ANTs;http://stnava.github.io/ANTs/,Avants et al., 2009), FSL (version 6.0,https://fsl.fmrib.ox.ac.uk/fsl/fslwiki/), and FSL FEAT (Woolrich et al., 2001).

To eliminate the drift from the data and to minimize potential effects of motion on the data, the fMRI time series were motion corrected. We established a pipeline utilizing the ANTs’ rigid registration. The time series were divided into individual volumes and registered to the first volume of the series. The realignment matrices were used to derive six motion parameters: three for translation and three for rotation. Next, we used the anatomical images to register the functional data. The standard registration tools are unsuitable for the zero echo time images, likely due to the different contrast produced by the zero echo time sequence compared with standard anatomical imaging. Although masking is frequently used to facilitate the challenges encountered in registration, the standard MRI masking tools proved to be inadequate for zero echo time images. Therefore, we generated brain masks on the anatomical images with a modified 2D version of a machine learning tool MedicDeepLabv3+ (Valverde et al., 2023) that was fine-tuned on the current dataset. The masked anatomical images were coregistered to a reference brain selected from the data using affine and nonlinear SyN registration, and the registration transformations were subsequently applied to the functional data. For the subject-wise functional maps, the time series were first high-pass filtered (0.01 Hz) and the autocorrelation was removed. The maps were calculated in FSL FEAT using a general linear model (GLM) with two predictors (for visual and auditory stimuli). The predictors were estimated by convolution of a boxcar function and a gamma-variate hemodynamic response function (HRF) by FSL FEAT with the following parameters: delay of the response 2.8 s, dispersion of the response 1.4 s. These parameters resemble the rodent HRFs reported in the literature (Nunez-Elizalde et al., 2022;Valjakka et al., 2023). As a way of accommodating for different responses observable in the awake and anesthetized animals, we used two different boxcar functions to convolute the predictors: 10 s (same as the stimuli duration) and 2 s (to explore fast responses, see Results section). The FSL Motion Outliers tool was used to detect time points that could have been affected by motion with exclusion criteria of DVARS (root mean square of intensity difference of volume N to volume N+1) above 30. The mean DVARS value was 19.6 ± 2.6 in anesthetized and 23.5 ± 3.3 in awake animals which resulted to 0.1% of volumes in anesthetized and 5.9% of volumes in the awake animals being labeled as outliers by the tool. These outliers along with the six motion parameters were used as a confounding explanatory variable in the GLM analysis as follows: for each outlier, a separate column of zeros was created with a value of 1 at the time point, which was labeled by the Motion Outliers tool. Additionally, six additional columns with the motion parameters were appended to the matrix. The FSL nonparametric permutation tool Randomise with the threshold-free cluster enhancement test statistic was used to estimate the group-level activation maps both under anesthesia and wakefulness, where p-values were corrected for family-wise error rate and values of p < 0.05 were considered significant (Salimi-Khorshidi et al., 2011;Winkler et al., 2014). Maps were overlaid on reference image that was obtained from all MT-weighted anatomical images averaged across all subjects.

To explore time course responses, the Allen Mouse Brain Atlas (Allen Institute for Brain Science,https://mouse.brain-map.org/static/atlas,Lein et al., 2006) was registered to the anatomical template using affine and nonlinear SyN registration. Regions of interest (ROIs) were selected in (i) the visual geniculate pathway: dorsal lateral geniculate nuclei (dLGN) and primary visual cortical area (VISp); (ii) visual extrageniculate pathway: superior colliculus (SC), lateral posterior thalamic nucleus (LP), higher-order visual areas (HVA, here we included postrhinal, anterolateral, rostrolateral, lateromedial, anterior, anteromedial, posterior, posterior medial, and laterointermediate visual areas); (iii) the auditory pathway: inferior colliculus (IC), medial geniculate complex (MGN), auditory cortical areas (AUD); (iv) polymodal association areas: subiculum complex (SUB), retrosplenial area (RS), temporal association area (TEa), medial frontal cortex (mFC, here we included anterior cingulate, prelimbic, and infralimbic areas), ectorhinal area (ECT); and (v) nonimage-forming midbrain visual area: pretectal nucleus (PRT). The six motion parameters were linearly regressed out from the time courses in a voxel-wise fashion, after which the mean time courses were extracted from each ROI. To explore the temporal difference between the awake and anesthetized animals, an individual t-test was conducted on the values of each volume from the mean time courses between the start of the stimulus and 11 volumes poststimulus onset (~19 s) for each ROI. The p-values were false discovery rate (FDR) corrected (Benjamini & Hochberg, 1995) and p-values <0.05 were considered significant. Subsequently, the mean responses across all animals and the confidence intervals of the mean values were plotted. Volumes identified as outliers using the FSL Motion Outliers tool were excluded from both the statistical analysis and the plots.

## 3 Results

Unprocessed MB-SWIFT fMRI images exhibited excellent coverage without distortions under the implant or around the ear canals, as shown inSupplementary Figure 1, indicating that we had acquired a high image quality suitable for whole-brain fMRI.

### 3.1 Responses to the visual stimuli in the visual pathways

Brain-wide responses to the visual stimulus were observed in the two major visual pathways: geniculate and extrageniculate pathways (Fig. 2). The responses in the subcortical areas of the extrageniculate pathway, superior colliculus (SC), and lateroposterior thalamic nucleus (LP) were similar in both shape and strength under anesthesia and in awake conditions. The higher-order visual cortical area (HVA) of the extrageniculate pathway showed a slightly steeper response in awake animals compared with the anesthetized mice. Similarly, the two nodes of the geniculate pathway, lateral geniculate nucleus of the thalamus (LGN), and primary visual cortical area (VISp) showed faster responses in the awake animals. Moreover, the shape of the response in the VISp in awake mice was very different when compared with both other ROIs and to the responses in anesthetized mice, exhibiting a biphasic response: that is, a short increase in the signal followed by an undershoot already during the stimulus. As a consequence, this led to the absence of the VISp in the activation maps obtained with conventional GLM analysis using a 10-s long stimulus block.

### 3.2 Responses to the auditory stimuli in the auditory pathway

**Fig. 3.**
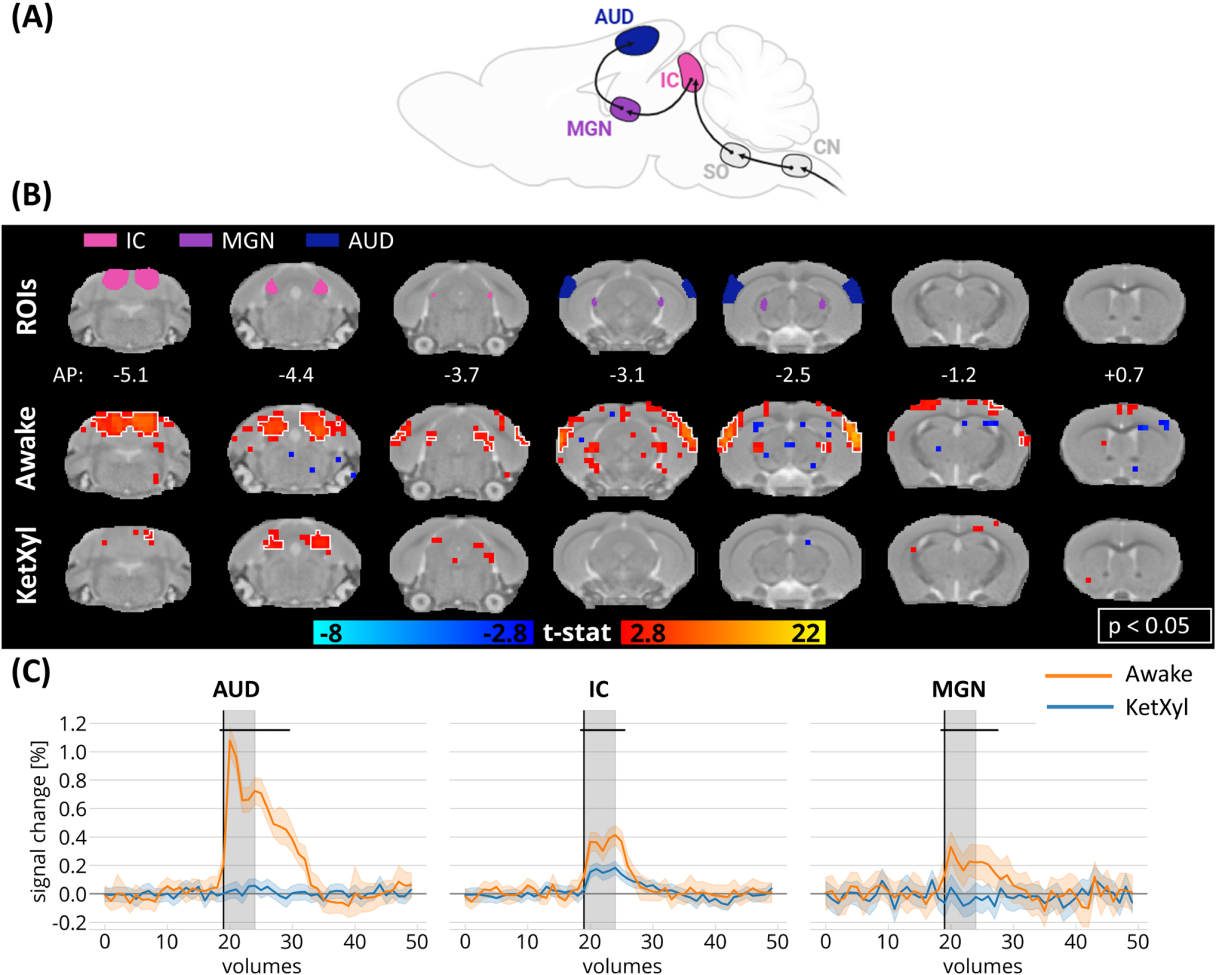
Activation maps and the time courses following auditory stimulation in awake and anesthetized animals in auditory pathway-relevant areas. (A) A simplified illustration of the auditory pathway in mice. (B) Regions of interest (ROIs) in the areas of the auditory pathway, and the group activation maps in awake (N = 23 measurements) and anesthetized animals (N = 20 measurements) in selected slices related to the visual pathway (white outline indicates p < 0.05, family-wise error corrected). (C) Mean time courses in the AUD, IC, and MGN in awake and anesthetized animals. Horizontal black lines represent the volumes where the two time series differed (independent volume-wise t-test, p < 0.05, FDR corrected, tested in volume numbers 19–30). Shaded plot: 95% confidence interval, the black vertical line marks the beginning of the stimulation and gray regions show the duration of the stimulus. AUD, auditory cortex; CN, cochlear nucleus; IC, inferior colliculus; KetXyl, ketamine–xylazine anesthesia; MGN, medial geniculate nucleus; SO, superior olivary complex. Partially created inhttps://www.BioRender.com.

Brain-wide responses to the auditory stimulus were observed in the auditory pathway in the wakefulness condition (Fig. 3). The strongest responses were seen in the inferior colliculus (IC), medial geniculate nucleus (MGN), and auditory cortex (AUD). The amplitudes of the cortical responses to the auditory stimuli were considerably higher than those observed in the cortical visual-evoked responses (1.1% in AUD compared with 0.4% in VISp). However, in the anesthetized group, the responses were substantially blunted in all analyzed ROIs. Only IC exhibited a statistically significant response, but it was still markedly below the values acquired in the awake group (0.2% in anesthetized vs. 0.4% in awake). These results point to the confounding effect of anesthesia on the auditory responses.

### 3.3 Responses to the visual stimuli in reflex-related and polymodal areas

**Fig. 4.**
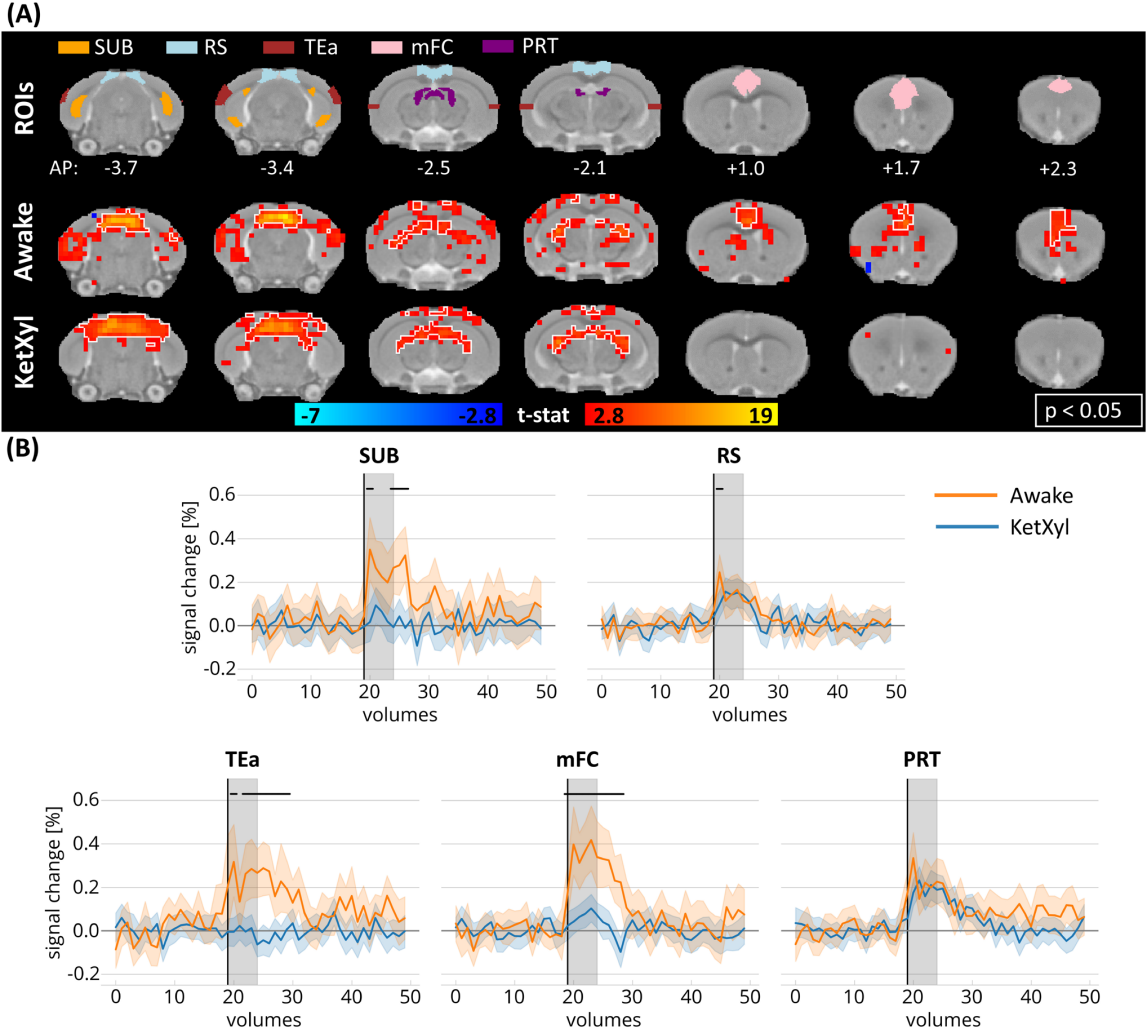
Activation maps and time courses following visual stimulation in awake and anesthetized animals in nonvisual-pathway areas. (A) Regions of interest in the polymodal and nonimage-forming visual areas and the group activation maps in awake (N = 23 measurements) and anesthetized animals (N = 20 measurements) in selected slices related to the selected ROIs (white outline indicates p < 0.05, family-wise error corrected). (B) Mean time courses in the selected nonvisual pathway areas. Horizontal black lines designate volumes where the two time series differed (independent volume-wise t-test, p < 0.05, FDR corrected, tested in volume numbers 19–30). Shaded plot: 95% confidence interval, the black vertical line marks the beginning of the stimulation and gray regions show the duration of the stimulus. KetXyl, ketamine–xylazine anesthesia; mFC, medial frontal cortex; PRT, pretectal nucleus; RS, retrosplenial cortex; SUB, subiculum; TEa, temporal association area.

Many brain areas outside the main visual pathways were also activated following the visual stimuli in both awake and anesthetized animals (Fig. 4). Both groups exhibited significant responses in the pretectal nucleus (PRT), which is responsible for the pupillary effect. Furthermore, we observed a potential activation in the polymodal association areas subiculum (SUB), retrosplenial cortex (RS), temporal association area (TEa), and medial frontal cortex (mFC) in awake animals. Though the activation in TEa and SUB was not statistically significant in the GLM maps, the t-values in these areas were above 3, and the mean time series were significantly different between awake and anesthetized mice. Moreover, the mean time series in these ROIs had similar shapes and only slightly lower amplitudes (0.3% of signal change) as in the main visual pathway ROIs. The mFC exhibited a strong response to the visual stimuli in the awake animals. Under anesthesia, only the RS included significant voxels among the polymodal areas. These observations suggest that higher sensory processing was activated by the visual stimuli in awake animals.

### 3.4 Responses to the auditory stimuli in polymodal areas

**Fig. 5.**
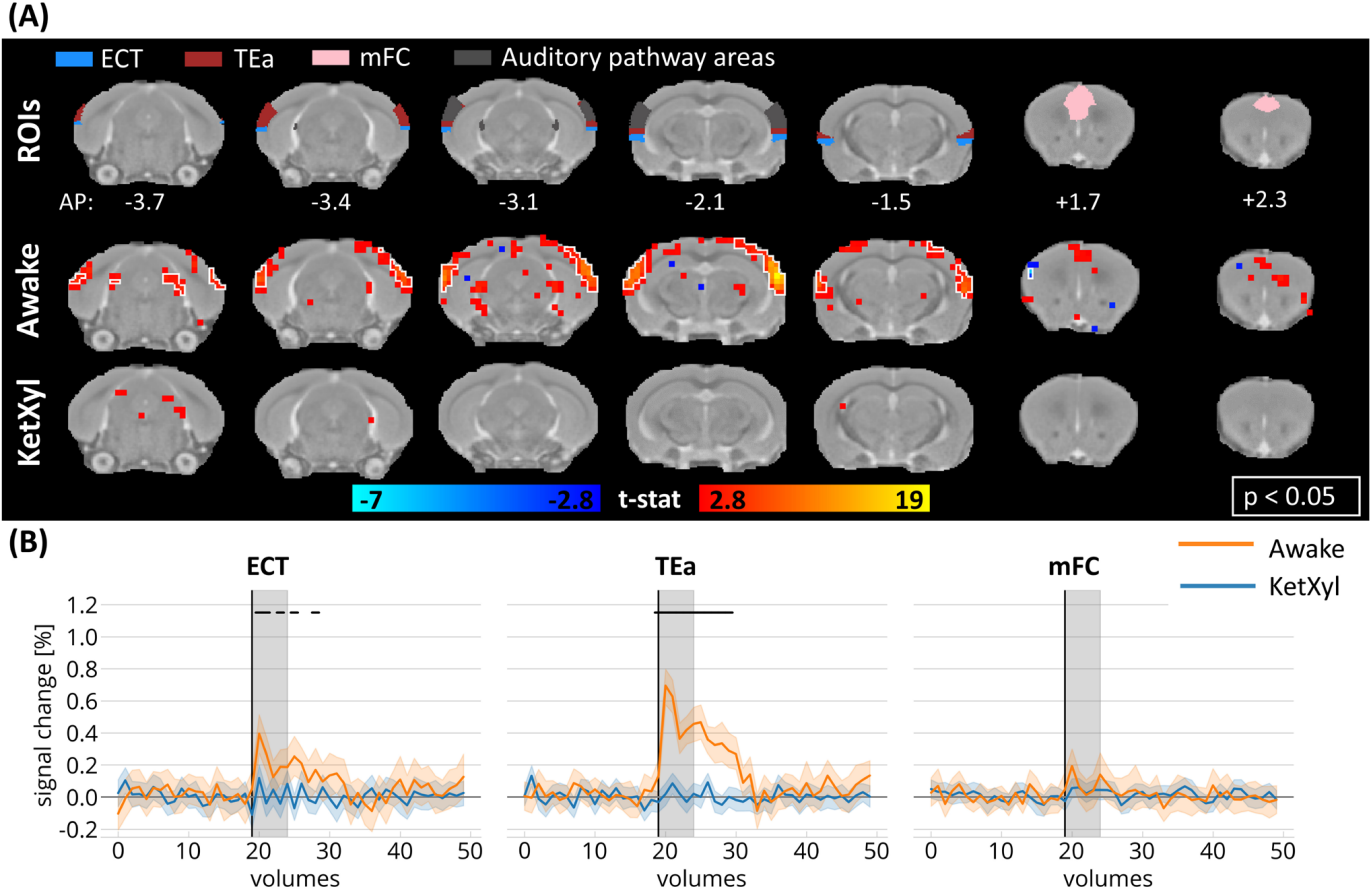
Activation maps and time courses following auditory stimulation in awake and anesthetized animals in nonauditory pathway areas. (A) Regions of interest in the polymodal areas and the group activation maps in awake (N = 23 measurements) and anesthetized animals (N = 20 measurements) in selected slices related to the selected ROIs (white outline indicates p < 0.05, family-wise error corrected). (B) The mean time courses in the selected nonvisual pathway areas. Horizontal black lines represent the volumes where the two time series differed (independent volume-wise t-test, p < 0.05, FDR corrected, tested in volume numbers 19–30). Shaded plot: 95% confidence interval, the black vertical line designates the beginning of the stimulation and gray regions show the duration of the stimulus. ECT, ectorhinal cortex; KetXyl, ketamine–xylazine anesthesia; mFC, frontal cortex; TEa, temporal association area.

Activations outside the main auditory pathway to the auditory stimuli were found exclusively in the awake animals (Fig. 5). Specifically, we detected responses in the TEa and ectorhinal cortex (ECT), both of which are structurally connected to the visual and auditory pathways (Nishio et al., 2018;Ohga et al., 2018). Potential responses, yet not statistically significant, were also found in mFC.

### 3.5 Responses estimated with a short boxcar GLM

**Fig. 6.**
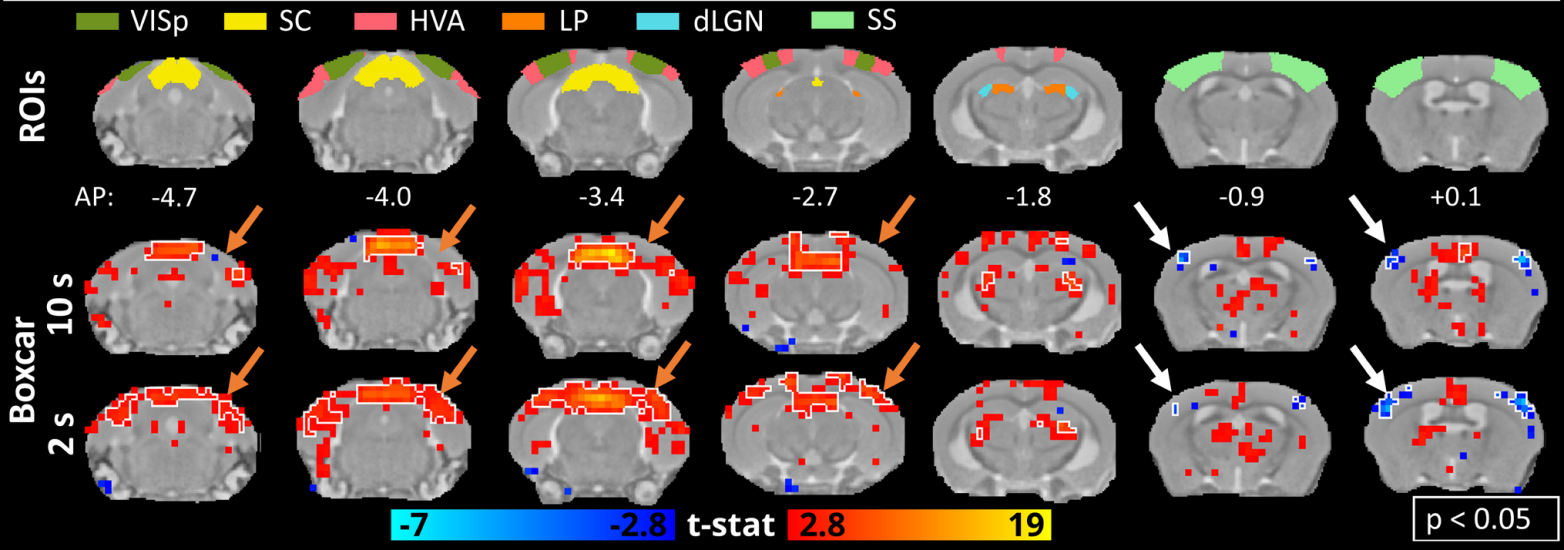
Comparison of activation maps to the visual stimulus estimated by HRF convoluted boxcar functions of two different lengths (2 and 10 s) in awake animals. Regions of interest in the areas of the visual pathway and the group activation maps (n = 23 measurements) in selected slices related to the visual pathway (orange arrows highlight the differences in the primary visual cortical areas, white arrows highlight the negative response in the somatosensory cortical areas, the white outline indicates p < 0.05, family-wise error corrected). dLGN, dorsal lateral geniculate nucleus; HVA, higher-order visual areas; LP, lateral posterior thalamic nucleus; SC, superior colliculus; SS, somatosensory cortical area; VISp, primary visual cortical area.

**Fig. 7.**
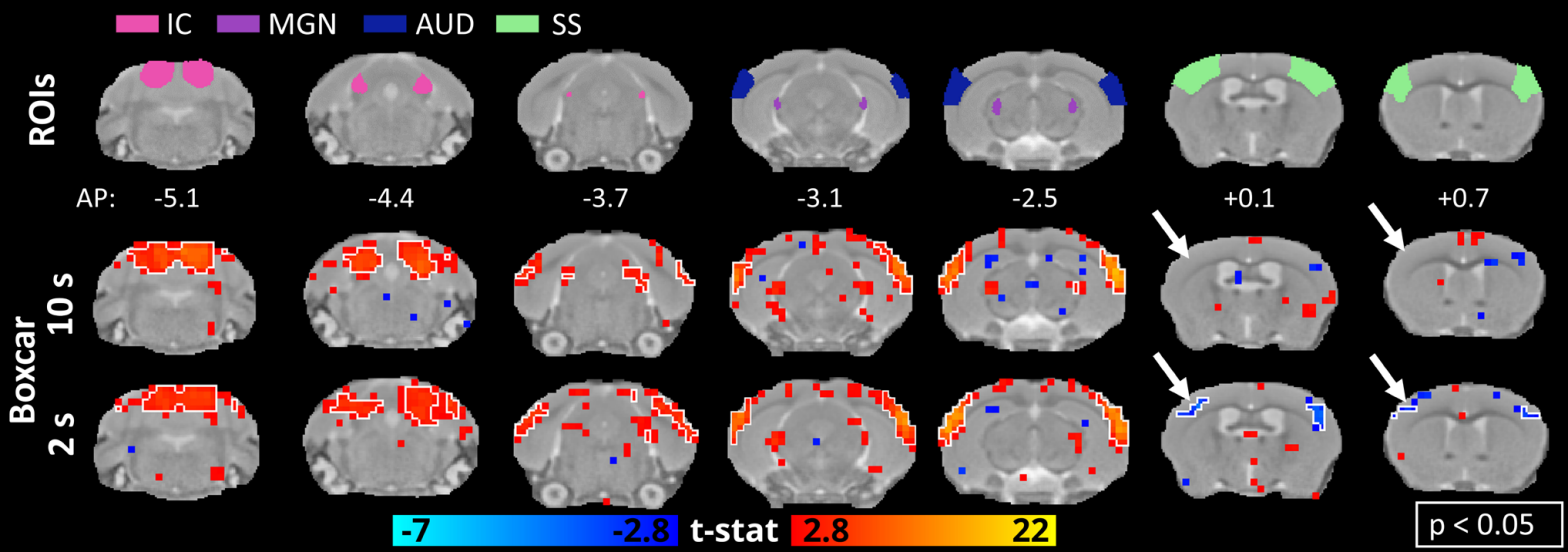
Comparison of activation maps with auditory stimulus estimated by HRF convoluted boxcar functions of two different lengths (2 and 10 s) in awake animals. Regions of interest in the areas of the visual pathway and the group activation maps (n = 23 measurements) in selected slices related to the auditory pathway (white arrows highlight the differences in the somatosensory cortical areas, the white outline indicates p < 0.05, family-wise error corrected). AUD, auditory cortical area; IC, inferior colliculus; MGN, medial geniculate nucleus; SS, somatosensory cortical area.

As a way of exploring the fast responses, activation maps were generated using a GLM with a 2-s boxcar predictor (Figs. 6and7). As there was no notable change in the anesthetized animals, only the maps of the awake mice are shown. In the activation maps following the visual stimulation, the most notable difference was the strong and significant response in VISp, which was missing with the classical estimation (orange arrows inFig. 6). In the auditory stimulation maps, the findings in the positive activations remained similar (Fig. 7). However, we observed significant negative responses in the somatosensory areas to both auditory and visual stimuli (time courses shown inSupplementary Fig. 3), which remained nonsignificant with the 10-s window in the auditory maps (white arrows inFig. 7). These results highlight the sensitivity of the GLM analysis to the selected predictor.

## 4 Discussion

In this work, we implemented zero echo time MB-SWIFT fMRI in minimally restrained awake mice. The MB-SWIFT approach allowed for whole-brain distortion-free and well-localized activation mapping in response to visual and auditory stimuli. Relatively quiet scanning, together with the habituation protocol, translated into only modest levels of animal motion, and awake mice were able to distinguish ambient auditory stimuli from the low background scanning noise.

The MB-SWIFT employs large bandwidths in all three directions, which in combination with zero echo time makes the approach tolerant to magnetic field inhomogeneities. Therefore, only quick manual adjustments of linear shims were needed in order to acquire good quality images. This led to short preparation and adjustment times, as there was no need for field-map-based shimming. Moreover, the MB-SWIFT approach is insensitive to susceptibility-induced image distortions (Laakso et al., 2021;Lehto et al., 2017;Paasonen et al., 2022). This made it possible to detect if there was an activation in rostral and inferior cortical areas such as the ectorhinal cortex, which have not been previously reported in mice. Moreover, no image distortions were visible around the head implant (Supplementary Fig. 1), which is an important factor when studying the superior parts of the cortex, such as the visual cortex as conducted in the current study. The robustness to the susceptibility-induced artifacts and small gradient switching-induced artifacts should make it possible to undertake combined fMRI and electrophysiological recordings (Paasonen et al., 2020).

As shown here, the quiet MB-SWIFT approach meant that we could apply a simple and straightforward auditory stimulation setup in head-fixed, unrestrained mice. This is crucial given the challenges posed by the loud acoustic noise of the standard EPI sequence. Recent studies in mice have attempted to mitigate this issue by attaching a plastic tube to the ear canal with paraffin or silicone (Blazquez Freches et al., 2018;Chen et al., 2020). This procedure, however, requires restraining the animals and prolongs the preparation and, therefore, time required for the induction of anesthesia, which substantially interferes with studies in conscious animals.

Another advantage of the zero echo time approach for fMRI data acquisition is its tolerance to motion artifacts. Although the animals were head fixed, which minimizes the impact of motion on image quality, motion correction was still incorporated into the preprocessing pipeline in this study to decrease the effect of residual motion and to mitigate any drift resulting from temperature-dependent gradient instability. Unlike standard fMRI sequences, the zero echo time sequence produces less contrast between white and gray matter. Additionally, the field of view includes nonbrain areas such as the ears and skin, which can exhibit significant motion during awake imaging. These combined factors often lead to difficulties in accurately registering individual volumes using standard tools. Here we established a preprocessing pipeline, which was fine-tuned on the current dataset. In most awake mouse studies, a framewise displacement has been used to exclude whole scans or individual volumes (Chen et al., 2020;Mandino et al., 2023). Instead of a metric relying on the motion correction parameters, we adopted an image intensity-based metric, which produces comparable exclusion criteria (Power et al., 2012). To further ensure that motion would have no effect on our results, motion parameters and the excluded volumes were used as confounding explanatory variables in the GLM analysis.

In the standard EPI approaches, the fMRI contrast relies on the blood oxygenation level-dependent (BOLD) effect, which depends on the echo time. Instead, in the zero echo time approach, the majority of fMRI contrast likely originates from changes in cerebral blood flow and volume that become visible due to the differences in magnetization saturation between tissue and inflowing blood during steady-state conditions, as occurs during the rapid radiofrequency pulsing in MB-SWIFT (Lehto et al., 2017). Despite the disparity in the origin of the fMRI contrast, our findings demonstrate that the MB-SWIFT technique yields comparable results with standard EPI methods. Here, we employed a similar anesthesia and stimulation protocol for visual-evoked fMRI as described byDinh et al. (2021). In both studies, the responses were slightly suppressed by anesthesia in the subcortical areas, whereas the cortical responses in awake animals exhibited an unusual biphasic temporal shape.

It has been claimed that the fMRI response shape in the primary visual cortical area is dependent on the visual stimulus frequencies both in awake and in anesthetized animals, exhibiting positive, negative, or biphasic profiles (Dinh et al., 2021;Hike et al., 2023;Niranjan et al., 2016). Despite the consistent use of a 5 Hz frequency across the studies in awake mice (Dinh et al., 2021;Hike et al., 2023), mixed results with both positive and biphasic shapes have been reported. The stimulation duration, however, differed across the studies, ranging from 4 to 10 s. These differences in stimulation train duration have been shown to affect the temporal characteristics of the fMRI response, as after 4 s of stimulation, the response amplitudes tend to notably decrease, likely reflecting the acute adaptation of sensory neurons (Chen et al., 2020). Therefore, we adopted the same stimulation paradigm as described by Dinh et al. (5 Hz, 10-s stimuli), and observed a similar biphasic response pattern (Dinh et al., 2021).

In addition to positive fMRI responses in several relevant brain regions, we detected negative responses in somatosensory areas to both visual and auditory stimuli. Interpreting these negative fMRI responses is challenging, as they may have multiple sources. The negative signal change may be related to a decrease in neuronal activity below the basal level (Cerri et al., 2024;Northoff et al., 2007;Shmuel et al., 2006). Alternatively, some researchers have attributed negative responses to neurovascular uncoupling (Goense et al., 2012;Staehr et al., 2023), or to a redistribution of blood flow from adjacent regions, also known as the “blood-stealing” effect (Puckett et al., 2014). Additionally, negative responses to loud noise stimuli in the somatomotor and motor areas have been reported in both human and mice (Hikishima et al., 2023;Kühn et al., 2004). This phenomenon is thought to be related to an audio-spinal reflex, although its exact source remains unclear (Hikishima et al., 2023;Kühn et al., 2004).

The GLM-based mapping is known to be sensitive to the selected predictor for the fMRI response (Schlegel et al., 2015). Commonly, fixed predictors have been employed in fMRI analysis. However, extensive variability in the temporal fMRI response has been reported across brain regions as well as between species, anesthetic protocols, and types of stimuli (Chen et al., 2020;Handwerker et al., 2012;Rosa et al., 2015;Schlegel et al., 2015). Here we also observed significant variation in the response to the stimuli, particularly in the cortical areas. Predictors in the GLM analysis are usually derived from the convolution of a boxcar function with the HRF, under the assumption of sustained positive activity following the stimuli. However, it is known that sensory activity adapts to a continuous input (Chen et al., 2020). Additionally, it has been shown that visual responses in the awake mouse cortex are dominated by inhibition (Haider et al., 2012;Nunez-Elizalde et al., 2022), leading to brief electrical responses followed by a peak of the opposite polarity (Haider et al., 2012). To accommodate both the fMRI responses variability and nonlinearity of the cortical responses, the common GLM-based analysis can include multiple basis functions for different conditions. However, this approach often lacks a biophysical foundation, and, therefore, its physiological interpretability is limited (Rosa et al., 2015). Moreover, the group-level analysis is not straightforward (Cignetti et al., 2016). Hence, we implemented a simpler approach and, in addition to the standardly generated GLM predictor by a boxcar function lasting for the duration of our sensory stimuli (10 s), we used a predictor generated by a shorter boxcar function (2 s). This allowed us to explore the cortical responses in awake animals, which exhibit fast and steep responses, while only slightly hindering responses in areas with slower responses, such as thalamic nuclei and colliculi.

Xylazine, a vasoconstrictive α2-adrenergic agonist, may impair the vasodilation of arterioles in response to neuronal activity, potentially affecting fMRI responses. This effect is counterbalanced by the vasodilatory properties of ketamine, a noncompetitive NMDA antagonist. However,Rakymzhan et al. (2021)reported cerebral vasodilation and elevated capillary flux under the combined ketamine and xylazine anesthesia in mice. These effects, along with other reported impacts of the anesthesia, such as reduced intracerebral heat production and decreased brain metabolism (Irwin et al., 2023), may contribute to the slightly slower responses to visual stimuli under anesthesia observed here. The precise mechanisms underlying the effects of anesthesia are, however, complex and not completely understood.

An interesting finding in the current work was the diminished responses to auditory stimuli in anesthetized animals, illustrating blunted processing of sound under the anesthesia protocol used. Other investigators have explored auditory-evoked fMRI responses in both awake and anesthetized rodents (Blazquez Freches et al., 2018;Chen et al., 2020;Cheung et al., 2012). These studies reported specific and robust thalamic and tectal activations to auditory stimuli in both conditions. While we observed similar responses in these areas in awake animals, anesthetized animals showed only a blunted activation of the inferior colliculus, and higher nodes along the auditory pathway were not activated. Additionally, this finding contrasts with recent studies showing that ketamine–xylazine anesthesia provides high-sensitivity and strong fMRI responses to somatosensory, visual, and olfactory stimuli (Dinh et al., 2021;You et al., 2021;Zhao et al., 2020). However, ketamine exhibits a dose-dependent impact on metabolism and cerebral blood flow, varying across brain regions (Franceschini et al., 2010). Ketamine has been shown to decrease blood flow and glucose utilization in the auditory structures (IC, MGN, AUD), while the visual system (SC, dLGN, LP, VISp) remained unaffected (Cavazzuti et al., 1987;Crosby et al., 1982). The auditory pathway does not appear to be entirely inhibited by ketamine–xylazine anesthesia, since electrophysiological cortical auditory-evoked potentials have been detected (Postal et al., 2022). Further research is, therefore, needed to fully understand the responsiveness of the auditory pathway under ketamine–xylazine anesthesia.

Another possible reason behind the lack of cortical responses to the auditory stimuli is the frequency of the stimulus. The dependence of the cortical responses on the frequency of the auditory stimuli has been shown both in humans and in mice (Postal et al., 2022;Rosburg & Mager, 2021). Higher frequencies tend to reduce cortical activation, while the thalamus responds reliably. Consequently, since we detected diminished responses in both the cortex and thalamus, our finding is unlikely due to a frequency-dependent effect.

It is also important to note that the mouse strain used here, C57BL/6, is known to succumb to an age-related decline of hearing, similar to the situation in humans (Malmierca & Ryugo, 2012;Mikaelian, 1979). The hearing loss pattern exhibits a gradual increase of the auditory brainstem response (ABR) threshold starting at 2 months of age (Mikaelian, 1979;Turner et al., 2005) and can lead to complete deafness at 24–28 months of age (Frisina et al., 2011). However, a recent study revealed that despite weak ABRs, which are typically taken as evidence of hearing loss, strong cortical responses were recorded (Rumschlag & Razak, 2021). Moreover, the hearing impairment has been connected to a loss of hair cells in specific regions of the inner ear, including the spiral ligament, the stria vascularis, and the basilar membrane (Ison et al., 2007). Given our observation of activation of the inferior colliculus, which is an ascending node from the inner ear, it is reasonable to assume that the suppressed auditory responses are more likely associated with anesthesia-related effects rather than age-related hearing loss, despite the age of the animals used here (8–12 months).

### 4.1 Limitations of the study

In this study, the main focus was on the cortical and subcortical responses and, therefore, also the size of the coil was selected to have higher sensitivity only within the cerebrum. Even though the brainstem was within the field of view, there were no significant auditory-evoked responses, which have been previously identified in anesthetized animals with a cryogenic coil (Blazquez Freches et al., 2018). A larger coil could be used to increase the SNR in the deeper brain areas and brainstem with a tradeoff for sensitivity and resolution.

Another limitation of this study was the use of earplugs, which may have affected the perception of auditory stimuli and the MRI sounds during the habituation. Additionally, the audio recording equipment used to record the fMRI sounds for the habituation had a limited sampling rate of 44.1 kHz, which did not fully capture the hearing range of mice (approximately 1–100 kHz,Koay et al., 2002).

A further concern in this study is the age of the animals, as middle-aged (8–12 months) mice were used. Although aging can affect both visual and auditory systems, the robust results in our awake measurements do not suggest a significant decline in sensory activation. Lastly, the brief exposure to isoflurane during the training and measurement days may affect brain function. However, isoflurane is rapidly eliminated from the body, with over 99% cleared from the brain within 5 min even after a 1-h of isoflurane exposure (Saab et al., 2010). While potential long-term effects of isoflurane cannot be completely ruled out (Stenroos et al., 2021), our results closely resemble awake measurements reported in the literature (Hike et al., 2023;Zeng et al., 2022), indicating no significant anesthetic impact on our findings. Moreover, we observed activation in the medial frontal cortex, similar to a study, where no isoflurane was used, indicating a similar conscious state of the animals (Hike et al., 2023). In the future, implementing an anesthesia-free habituation protocol could help eliminate any potential lingering effects of anesthesia.

## 5 Conclusions

To conclude, we have pioneered a novel fMRI approach which can be applied in awake mice utilizing an acoustically quiet and artifact-free zero echo time MB-SWIFT pulse sequence. With this novel approach, we detected whole-brain fMRI responses in awake animals both in sensory pathways and in higher-order association areas. While ketamine–xylazine anesthesia was found to be a credible alternative to awake imaging for studying the visual system, in contrast this form of anesthesia strongly suppressed responses in the auditory system. The new approach paves the way for more complex behavioral sensory fMRI designs that hold the promise of improving our understanding of sensory processing.

## Supplementary Material

Supplementary Material

## Data Availability

The data and the codes used here are openly available here:https://doi.org/10.23729/f25d01ae-1b27-4395-8446-2dfd37d80a76.
