# Supplementary material for: Whole-brain responses to visual and auditory stimuli in anesthetized and minimally restrained awake mice using quiet zero echo time fMRI
Source: Imaging Neurosci (Camb). 2024 Dec 5;2:imag-2-00384. doi: 10.1162/imag_a_00384 (PMC12330383; doi:10.1162/imag_a_00384)
Supplement: Supplementary Material [file imag_a_00384-supp.pdf]

Supplementary materials

Supplementary table 1: Habituation protocol and timeline of the MRI and timeline of the MRI experiments. Day of the experiment, where X is 21-63 days (3-9 weeks); Duration: the total duration of the session (pre-training phase) or the duration, when the anesthesia was lowered to 0% (training phase); Animal handling: manual handling in the beginning of the session to familiarize the animal with the handler; Animal freely in holder: the mouse was allowed to explore and become familiarized with the MRI holder; Hand-held restraint: the headpost was held for short intervals of time; Restraint in holder: animal was placed in the holder and the headpost was secured; MRI sound: the prerecorded sound of the fMRI sequence was played through a loudspeaker. Earplugs: mouldable silicone earplugs were inserted into the ear canals.

	**Pre-training phase**					**Training phase**									**MRI (awake)**			**MRI (anesthesia)**		
Day of the experiment	1	2	3	4	5	8	9	10	11	12	15	16	17	18	19	22	24	X	X+7	X+14
Duration [min]	10	10	10	10	10	5	10	15	20	25	25	25	25	25	-	-	-	-	-	-
Animal handling	+	+	+	+	+	-	-	-	-	-	-	-	-	-	-	-	-	-	-	-
Animal freely in the holder	-	+	+	+	+	-	-	-	-	-	-	-	-	-	-	-	-	-	-	-
Hand-held restraint	-	-	+	+	+	-	-	-	-	-	-	-	-	-	-	-	-	-	-	-
Restraint in holder	-	-	-	-	-	+	+	+	+	+	+	+	+	+	+	+	+	+	+	+
MRI sound [%]	-	-	-	20	20	40	60	80	100	100	100	100	100	100	-	-	-	-	-	-
Earplugs	-	-	-	-	-	-	-	+	+	+	+	+	+	+	+	+	+	+	+	+
Stimuli	-	-	-	-	-	-	-	-	-	-	-	+	+	+	+	+	+	+	+	+

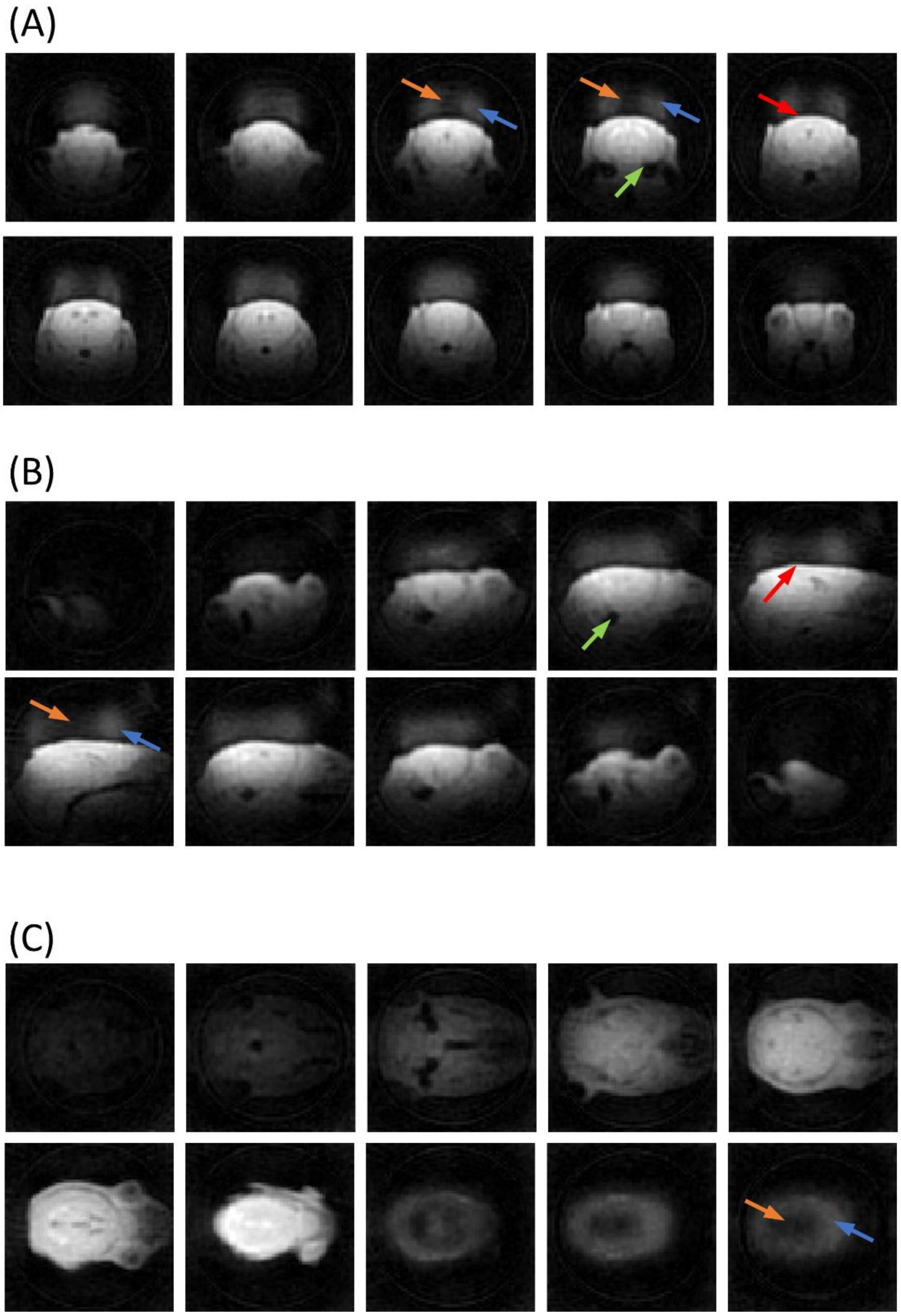

Supplementary Figure 1: A representative example of raw MB-SWIFT fMRI images in the (A) anterior-posterior, (B) lateral, and (C) inferior-superior directions. The implant, which includes a plastic headpost (orange arrows) surrounded by dental cement (blue arrows), preserves the integrity of the underlying cortex (red arrows). Additionally, the images were not sensitive to distortions around the ear cavities (green arrows), which are typically encountered in EPI images.

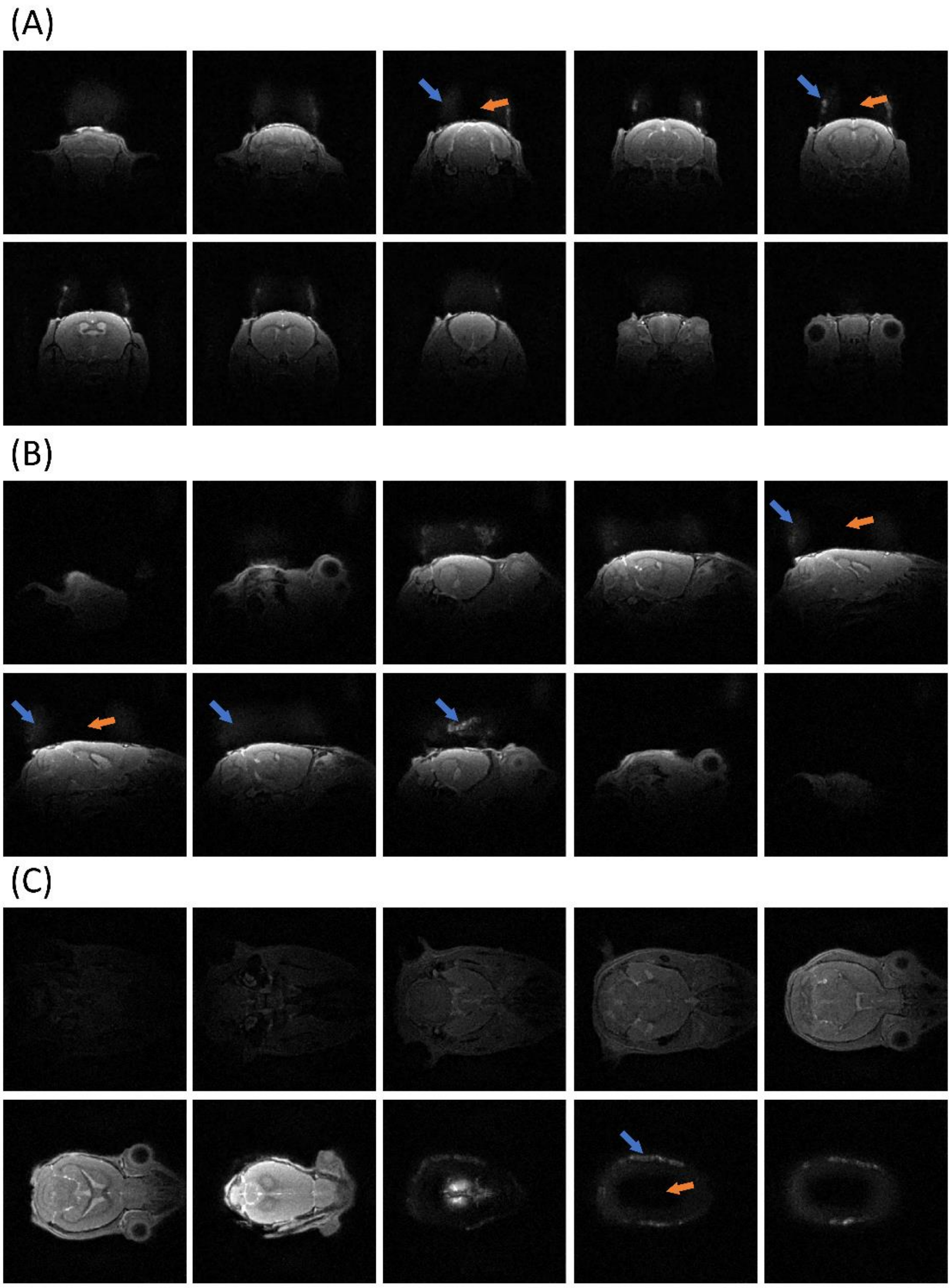

Supplementary Figure 2: A representative example of raw MT-weighted MB-SWIFT fMRI images in the (A) anterior-posterior, (B) lateral, and (C) inferior-superior directions. The images were denoised using an adaptive non-local means denoising (Manjon et al. 2010).

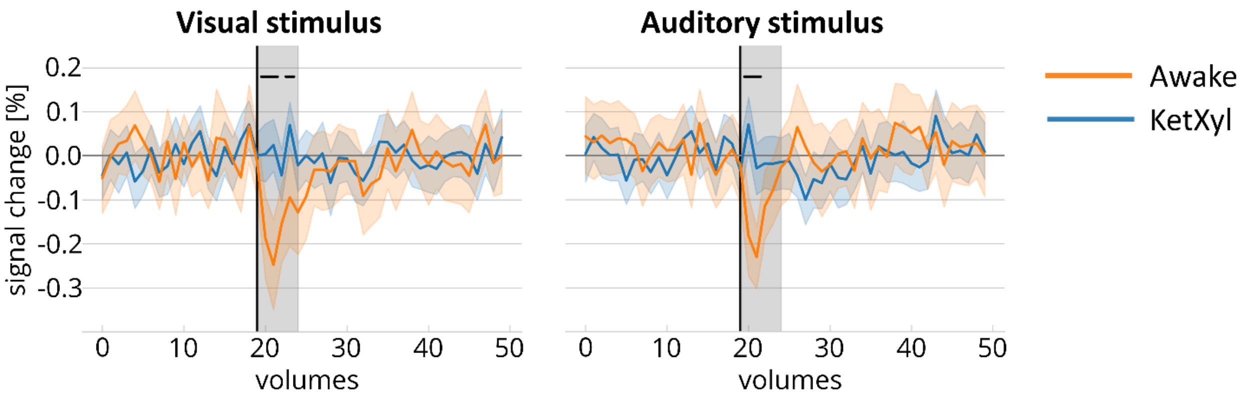

.

Supplementary Figure 3: The mean time courses in a part of the somatosensory cortical area. Horizontal black lines represent the volumes where the two time series differed (independent volume-wise t-test, p<0.05, FDR-corrected, tested in volumes number 19-30). Shaded plot: 95% confidence interval, the black vertical line designates the beginning of the stimulation and the grey regions show the duration of the stimulus. KetXyl, ketamine-xylazine anesthesia.

References:

1. J. V. Manjon, P. Coupé, L. Martí-Bonmatí, D. L. Collins, M. Robles. Adaptive Non-Local Means Denoising of MR Images with Spatially Varying Noise Levels*. Journal of Magnetic Resonance Imaging, 31(1):192–203, 2010.*
